# Memory stem CD8^+^T cells in HIV/Mtb mono- and co-infection: characteristics, implications, and clinical significance

**DOI:** 10.3389/fcimb.2024.1485825

**Published:** 2024-12-10

**Authors:** Jing Xiao, Fuchun Wang, Hongxia Yan, Bo Wang, Bin Su, Xiaofan Lu, Tong Zhang

**Affiliations:** ^1^ Beijing Key Laboratory for HIV/AIDS Research, Sino-French Joint Laboratory for HIV/AIDS Research, Clinical and Research Center for Infectious Diseases, Beijing Youan Hospital, Capital Medical University, Beijing, China; ^2^ Department of Respiratory Medicine, Beijing Fengtai Hospital of Integrated Traditional and Western Medicine, Beijing, China

**Keywords:** HIV, *Mycobacterium tuberculosis*, CD8 + T cells, memory stem T cells, exhaustion

## Abstract

Human immunodeficiency Virus (HIV) and *Mycobacterium tuberculosis* (*Mtb*) co-infection presents a significant public health challenge worldwide. Comprehensive assessment of the immune response in HIV/*Mtb* co-infection is complex and challenging. CD8^+^T cells play a pivotal role in the adaptive immune response to both HIV and *Mtb*. The differentiation of CD8^+^T cells follow a hierarchical pattern, with varying degrees of exhaustion throughout the process. Memory stem T cells (T_SCM_ cells) is at the apex of the memory T lymphocyte system, which has recently emerged as a promising target in immunotherapy. In this context, we discuss the alterations of CD8^+^T_SCM_ cells in HIV/*Mtb* mono- and co-infection, their implications and clinical significance, and potential for improving immunotherapy.

## 1 Introduction

Human immunodeficiency Virus (HIV) and *Mycobacterium tuberculosis* (*Mtb*) co-infection has been an urgent public health problem worldwide. Coinfection with HIV accelerates the progression of *Mtb* infection and exacerbated its severity (Ajayi et al., 2022; World Health Organization, 2020; Seyoum et al., 2022; Sultana et al., 2021). Nowadays, tuberculosis (TB) remains the leading cause of death among people living with HIV (PLWH). According to the latest data released by the World Health Organization (WHO), TB accounts for approximately 27% of AIDS-related deaths worldwide (World Health Organisation, 2023). What’s more, comprehensive assessment of immune response turns to be complicated and challenging in HIV/*Mtb* co-infection (Manna et al., 2020).

Although CD4^+^T cells are traditionally regarded as the primary IFN-γ producers in TB, which is pivotal in host defense against *Mtb*, vaccine trial setbacks suggest a need for reevaluation and exploration of alternative immune targets. Recently, protective role of CD8^+^T cells was revealed in early control of *Mtb* infection (Winchell et al., 2023). At the same time, an extensive body of evidence indicates that CD8^+^T cells play a fundamental role in the adaptive immune response to HIV. Exploration of CD8^+^T cells as alternative immune targets is prospective, and figuring out the characteristics of CD8^+^T cells in mediating cellular immunity in HIV/*Mtb* co-infection would offer a rationale for harnessing long-term control to combat disease.

Memory stem T cells (T_SCM_ cells), a newly defined memory T cells endowed with extreme longevity and robust potential for immune reconstitution (Gattinoni et al., 2017). T_SCM_ cells are commonly generated during natural immune responses against foreign pathogens. Though not fully characterized, work in the context of HIV or *Mtb* infection has shown the pertinence between CD8^+^T_SCM_ cells and both diseases, implying distinct role of this subsets in chronic infection. What’s more, functionally distinct from other memory subsets of T cells, CD8^+^T_SCM_ cells demonstrate a promising outlook in immunotherapy (Marraco et al., 2015). Taking CD8^+^T_SCM_ cells as a starting point to explore its regulatory mechanisms in HIV/*Mtb* co-infection may contribute to enhancing the efficacy of vaccines and adoptive T-cell therapies for *Mtb* infection in the context of HIV co-infection.

In this review, we discuss the alteration of CD8^+^T_SCM_ cells in HIV/*Mtb* co-infection, implications and clinical significance, and its potential for improvement of immunotherapy. Given the limited research on CD8^+^T_SCM_ cells in HIV/*Mtb* co-infection, we initially examined the patterns of CD8^+^T cells in both HIV and *Mtb* mono-infections as well as co-infections, aiming to gain insights that could contribute to the study of CD8^+^T_SCM_ cells.

## 2 Partial recoveries of CD8^+^T_SCM_ cells under ART in HIV infection

In the absence of antiretroviral therapy (ART), the initial burst of HIV replication is characterized by an increase in viral load in blood. Subsequently, the viral load decreases, and this temporal shift coincides with an elevation in HIV-specific CD8^+^T cells, which is crucial for eliminating HIV-infected T cells (Walker et al., 1987). In most people living with HIV without ART treatment, HIV-specific CD8^+^T cells maintain dysfunctional during chronic HIV infection, because of continuous HIV antigen burden (Trautmann et al., 2012). Recently, research has reported that long-term ART initiated in Fiebig stage I prevents residual dysfunction of HIV-specific CD8^+^T cells (Takata et al., 2022), but most patients are unable to initiate ART treatment promptly, and residual dysfunction of HIV-specific CD8^+^T cells maybe a common phenomenon among HIV patients. In HIV-infection, T cells specific to other pathogen also manifest immune abnormalities. Latent viruses, such as cytomegalovirus (CMV) and Epstein-Barr virus (EBV), reactivate more frequently during HIV-1 infection due to the depletion of T cells that control viral replication (Walton et al., 2013). It has been observed that perforin expression in EBV- and CMV-specific CD8^+^T cells is reduced in HIV-infected patients, and this defect is accompanied by a lower expression of granzyme B (Zhang et al., 2003). In HIV/HCV co-infection, HCV-specific CD8^+^T cells co-express Tim-3 and PD-1 were in significantly higher frequencies and positively correlated with a clinical parameter of liver disease progression (Vali et al., 2010). Despite ART-induced viral suppression, alterations of CD8^+^T cells from HIV-infected patients include: 1) persistently increased absolute counts but impaired proliferative capacity (Helleberg et al., 2015; Gaiha et al., 2014); 2) defect in cytotoxic program (Perdomo-Celis et al., 2019b); 3) persistent immune activation and systemic inflammation (Hunt et al., 2003; Olson et al., 2021);4) defect in differentiation into functional cells (Takata et al., 2023); 5) persistent exhausted status (Trautmann et al., 2006; Jin et al., 2010; Wang et al., 2020).

**Figure 1:**
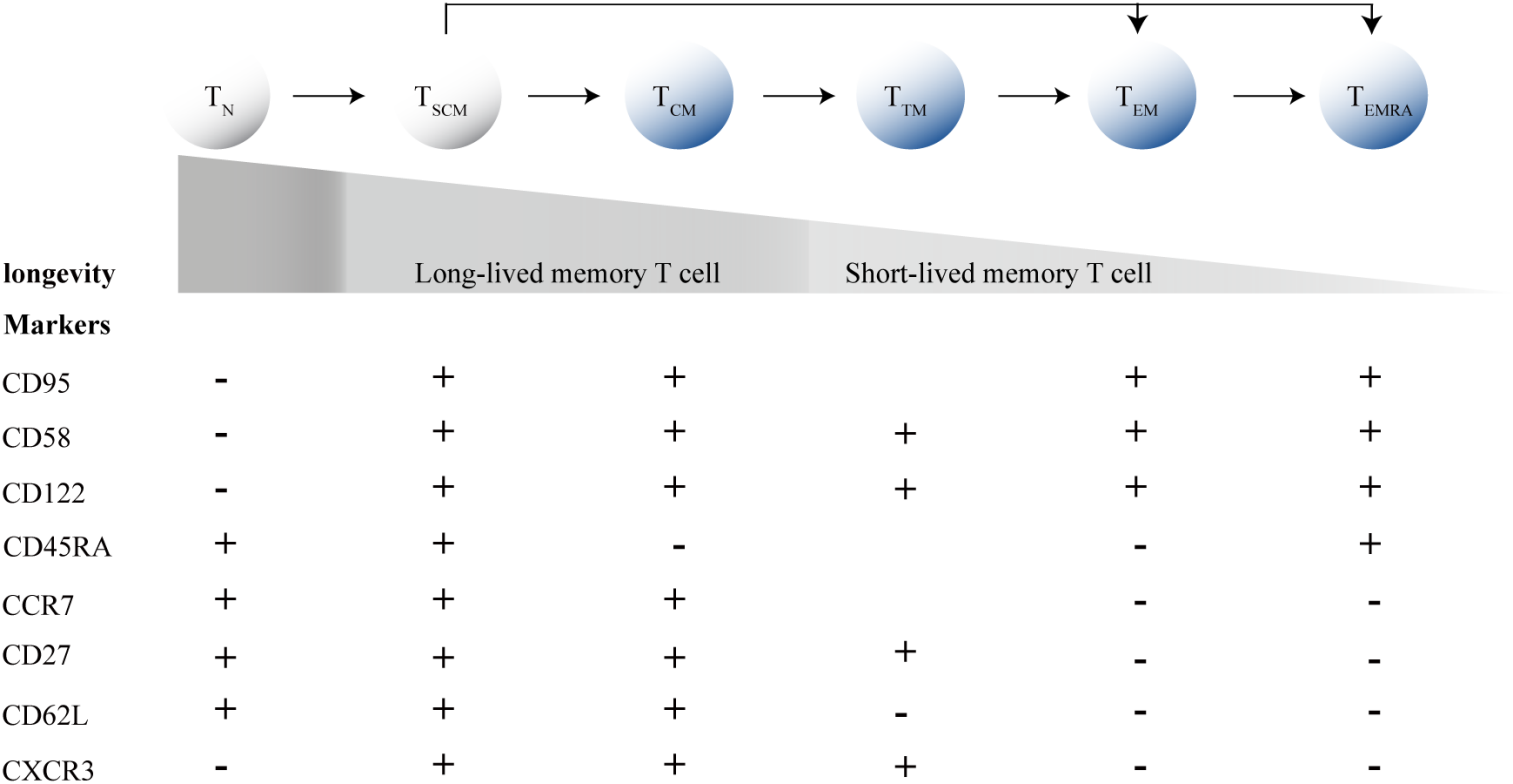
T cell differentiation process and marker expression profiles. T_N_, Naive T cells; T_SCM_ cells, memory stem T cells; T_CM_ cells, central memory T cells; T_EM_ cells, effector memory T cells; T_TM_ cells, transitional memory T-cells; T_EM_ cells, effector memory T cells; T_EMRA_, terminally differentiated effector T cells.

Furthermore, HIV significantly impacts the differentiation of CD8^+^T cells. CD8^+^T cells can differentiate into memory and effector subsets, with T_SCM_ cells and central memory T cells (T_CM_ cells) acting as “stem-like” precursors within the memory subset. Between the two types of T cell subsets, T_SCM_ cells are phenotypically defined as naive T cells (T_N_ cells) by the expression of T_N_ cell markers, such as CD45RA and CCR7, but distinguishable from T_N_ cells by two memory T cell markers: CD95, CD58 and CD122, and excelling in typical T_CM_ cells cell traits, but less phenotypically differentiated than T_CM_ cells and are overall less frequent (**Figure 1**) (Lugli et al., 2013; Gattinoni et al., 2011). Thus, they represent cells at an intermediate state of differentiation between T_N_ and T_CM_ cells. Commonly, after antigen priming, T_N_ cells progressively differentiate into diverse memory T cell subpopulations, and ultimately into terminally differentiated effector T cells (**Figure 1**). During acute HIV infection, memory CD8^+^T cells are driven toward a more terminally differentiated status, along with a decrease frequency of long-lived T cell subsets, including T_SCM_ cells and T_CM_ cells, promoting the differentiation of CD8^+^T cells with short-lived transitional memory (T_TM_ cells) and effector memory (T_EM_ cells) subsets (Takata et al., 2022). T_SCM_ cells and T_CM_ cells are the fount to sustain persistent CD8^+^T cell responses, and a failure to generate proliferation-competent precursor cells in chronic infections results in the collapse of the T cell response (Zehn et al., 2022).

As minimally differentiated cells at the apex of the hierarchical system of memory T lymphocytes, T_SCM_ cells endowed with the stem cell-like ability to self-renew and had multipotent capacity to reconstitute the entire spectrum of memory and effector T cell subsets (Gattinoni et al., 2009; Ahmed et al., 2016). What’s more, T_SCM_ cells have an exceptional capacity to persist long term proved in HIV infection (Vigano et al., 2015). HIV-specific CD8^+^T_SCM_ cells represent a long-lasting component of the cellular immune response to HIV-1 and are detectable during all stages of HIV-1 infection (Vigano et al., 2015). In HIV-exposed seronegative individuals and HIV patients with treatment interruption, count and frequency of HIV-specific CD8^+^T cells with stem cell-like phenotypes elevated, which implies the antiviral role of T_SCM_ cells in control of HIV infection (Ponnan et al., 2021; Sachdeva et al., 2023). Indeed, natural preservation of CD8^+^T_SCM_ cells in the setting of untreated HIV-1 infection is associated with improved viral control and immune reconstitution (Ribeiro et al., 2014). In the CD8^+^T cell compartment of ART-naive pediatric slow progressors, an enrichment of T_SCM_ cells were identified, whereas pediatric progressors and viremic adults had a terminally exhausted population (Vieira et al., 2023).

Although ART can result in an undetectable viral load in peripheral blood plasma and significantly reduce the HIV reservoir and CD8^+^T cell responses after 2 years of ART, the persistent viral reservoir continues to impact the differentiation status of HIV-specific CD8^+^T cells (Takata et al., 2023). The proportion of CD8^+^T cells increase during the acute phase of HIV infection, but there is a decrease in T_N_ cells (Perdomo-Celis et al., 2019a). Defined by traditional T cell subset markers such as CD45RA and CD62L or CCR7, it did not distinguish T_SCM_ cells from T_N_ cells, meaning that the T_N_ population in earlier studies actually included both T_N_ and T_SCM_ cells. As the precursor to other memory T cells, it can be speculated that the proportion of T_SCM_ cells also decreases following viral stimulation during the acute phase of HIV infection. Indeed, a decline in frequency of T_SCM_ cells can already be observed during Fiebig stages III and IV of HIV infection (Takata et al., 2022). TCF-1, a transcription factor important for self-renewal capacity, marks a population of stem-like CD8^+^T cells and sustain the immune response to chronic viral infections (Escobar et al., 2020; Utzschneider et al., 2016). The decrease in TCF-1 expression levels and the increase in PD-1 expression levels in CD8^+^T cells during HIV infection suggest that the loss of stem-like CD8^+^T cells including T_SCM_ cells in HIV infection may be due to the functional impairment, specifically their sustained proliferative capacity and self-renewal ability.

Actually, ART had an immune restorative effect on CD8^+^T_SCM_ cells (Tuluc et al., 2017), and the earlier the ART timing, the better effect the recovery. ART initiation in acute HIV infection promoted the persistence of HIV-specific CD8^+^T_SCM_ cells, with high expansion and cytotoxic capacity, and mitigatory activated/exhausted phenotype, whereas ART initiation in chronic HIV infection led to more differentiated HIV-specific CD8^+^T cells with a higher combined frequency of short-lived T cells (Takata et al., 2022; Salido et al., 2018; Tartaro et al., 2022). In patients with ART, the proportion of CD8^+^T_SCM_ cells rises to the level of healthy controls after 144 weeks of treatment (Song et al., 2017). On the contrary, the frequency of CD8^+^T_SCM_ cells was decreased in all individuals with chronic, untreated HIV-1 infection (Ribeiro et al., 2014). Initiation of ART recovered the expression of TCF-1, but HIV-specific CD8^+^T cells from people treated during Fiebig stage I expressed significantly higher TCF-1 compared with people treated during Fiebig stages III and IV (Takata et al., 2022). Although current HIV treatment guidelines emphasize initiating ART as early as possible, detecting and treating PLWH at Fiebig stage 1 remains very challenging, thus, functional impairments in T_SCM_ cells exist in most HIV-infected individuals.

## 3 Involvement of CD8^+^T_SCM_ cells in *Mtb* infection

The changes in the proportion of CD8^+^T cells in *Mtb* infection are still inconclusive. In various studies, the observed results regarding changes in the proportion of CD8^+^T cells due to *Mtb* infection are not consistent. Discrepancies exist in the alterations of the overall proportion of CD8^+^T cells across various studies on *Mtb* infection. Kudryavtsev I et al. found no differences in the CD8^+^T cells frequencies in peripheral blood between patients with pulmonary TB and healthy controls (Kudryavtsev et al., 2023). However, Chávez-Galán et al. found that TB patients had a higher frequency of CD8^+^T cells from same type samples (Chávez-Galán et al., 2019). When it comes to *Mtb*-specific CD8^+^T cells populations, divergent opinions persist across various studies. Cheryl L. Day et al. and Virginie Rozot et al. found no difference in percentage of *Mtb*-specific CD8^+^T cells between TB and latent *Mtb* infection (LTBI) patients (Day et al., 2014; Rozot et al., 2015). But subsequent studies found that TB patients had increased frequencies of *Mtb*-specific CD8^+^T cells, compared with LTBI (Pollock et al., 2013; Azgomi et al., 2022; Caccamo et al., 2015). Heterogeneity in results from different researches may be attributed to different methods employed to generate *Mtb*-specific CD8^+^T cell, including marking T cell by *Mtb* proteins tetramer, stimulating T cell by ESAT-6 and CFP-10 or stimulating by peptides pools covering a variety of antigen of *Mtb*. Considering immune response of *Mtb*-specific CD8^+^T cells is associated with *Mtb* and were predominantly found in patients with active TB compared to those with LTBI (Prezzemolo et al., 2014; Lancioni et al., 2019; Rozot et al., 2013), the observation that the proportion of *Mtb*-specific CD8^+^T cells increased in *Mtb* infection may be more reflective of the actual scenario. Considering the absolute changes, active TB led to reduced levels of CD3^+^ and CD4^+^T cells, but increased levels of CD8^+^T cells, confirming the rise in the CD8^+^T cells proportion (Li et al., 2020). Methodologically, it is more reliable to generate *Mtb*-specific CD8^+^T cell by using peptides pools covering a variety of antigen rather than just ESAT-6 and CFP-10, which is consistent with previous studies that adequate antigen is a prerequisite for the generation of *Mtb*-specific CD8^+^T cells (Lancioni et al., 2012).

What’s more, the proportion of CD8^+^T cells are subject to dynamic changes in *Mtb* infection. Compared with persons with LTBI, *Mtb*-specific CD8^+^T cells from TB diseased patients had significantly higher expression of Ki67, which is a cellular proliferation marker (Kudryavtsev et al., 2023). Indeed, TB patients had increased frequencies of *Mtb*-specific CD8^+^T cells compared with LTBI (Day et al., 2011). Significant changes in Ki67 expression of *Mtb*-specific CD8^+^T cells were observed two months after the initiation of anti-TB chemotherapy, accompanied by decreased frequency of *Mtb*-specific CD8^+^T cells, and to the comparable levels as healthy controls at the end of treatment (Day et al., 2014; Li et al., 2020; Day et al., 2011; Nyendak et al., 2013). The above study indicates that CD8^+^T cells are critical immunological players throughout the course of *Mtb* infection, including LTBI, active TB, and during anti-TB treatment.

The immune response of CD8^+^T_SCM_ cells in *Mtb* infection shares many similarities with their precursor cells—CD8^+^T cells. *Mtb*-specific T_SCM_ cells were not detected in a negative QuantiFERON Gold In-Tube (QFT) test persons. After QFT conversion, frequencies of T_SCM_ cells increased to measurable levels and remained detectable thereafter, suggesting that primary *Mtb* infection induces T_SCM_ cells (Mpande et al., 2018; Sun et al., 2024). For individuals with LTBI, the host sustains a complex interaction with *Mtb* through the regulation of nutrient availability, as well as the innate and adaptive immune responses, including the dynamic shifts in T_SCM_ cells. This relationship can lead to the reversion of tuberculin skin tests (TSTs) and IFN-γ release assays (IGRAs) from positive to negative in some individuals (Drain et al., 2018). Among those with measurable responses, lower proportions of T_SCM_ cells were observed in reverters, defined as adolescents with two positive QFT tests followed by two negative QFT tests 6 months apart, compared with non-converters (Mpande et al., 2021). These findings suggest that T_SCM_ cells may not only be involved in the immune response induced by *Mtb* but also play a role in well-controlled or previously cleared *Mtb* infections.

## 4 CD8^+^T_SCM_ cells exhaustion in *Mtb* infection

In the process of *Mtb* infection, CD8^+^T cells play a role in fighting against *Mtb*, simultaneously, progressive impairment of *Mtb*-specific CD8^+^T cell responses was observed with increasing *Mtb* load (Day et al., 2011). *Mtb*-specific T cell population displaying significant bioenergetic insufficiencies, declining mitochondrial health, and limited cytokine production, all early indicators of T cell exhaustion during *Mtb* infection (Russell et al., 2019). Indeed, T cell exhaustion is a significant feature of *Mtb* infection, revealed by a single-cell transcriptome atlas, the immune landscape in severe TB patients was characterized by widespread immune exhaustion in CD8^+^T cells (Wang et al., 2023). Successful anti-TB treatment results in restoration of *Mtb*-specific CD8^+^T cell function, the proportions can return to normal levels, but its limited proliferative function, a part of T cell progressive development exhaustion, may not be fully restored with the progress of treatment (Day et al., 2014). As expected by exhausted T cells, CD8^+^T cells display reduced production of cytotoxic granule molecules expression levels of perforin and granulysin in *Mtb* infection (Shen et al., 2023), and increased expression levels of suppressive cytokines, such as IL-10 (Jalbert et al., 2023). Furthermore, numerous previous studies have confirmed that through the detection of increase exhaustion markers on *Mtb*-specific CD8^+^T cells, such as CD57, PD-1, CTLA-4, KLRG-1, BATF, NKG2A in *Mtb* infection (Day et al., 2014; Russell et al., 2019; Liu et al., 2019; Shen et al., 2023). Antibody-mediated blockade of inhibitory receptor signaling pathways has been shown to enhance *Mtb*-specific T cell function (Shen et al., 2016). Checkpoint blockade immunotherapy in the treatment of *Mtb* infection is promising to promote control of disease. But in the subset of granulomas with ongoing caspase 1 activation, PD-1 blockade resulted in the exacerbation of *Mtb* infection, accompanied by the significantly enhanced expansion and function of *Mtb*-specific CD8^+^T cells in granulomas, though there were no definite conclusions regarding the contributions of CD8^+^T cells to the detrimental outcome of PD-1 blockade (Kauffman et al., 2021). McCaffrey et al. showed that the few PD-1-expressing lymphocytes present are largely concentrated in neighboring tertiary lymphoid structures (TLSs) (Mccaffrey et al., 2022). This distribution may help explain how PD-1 blockade exacerbates immunopathology by activating TLS-resident and peripheral T cells, while failing to engage granuloma T cells. Furthermore, PD-L1 blockade is another widely used strategy in anti-PD-1/PD-L1 immunotherapy. Compared with PD-1 blockade, while PD-L1 blockade also enhances CD8^+^T cells function, it may have a broader impact on the microenvironment by affecting other immune cells that express PD-L1. This can lead to a more complex modulation of the immune response, potentially enhancing overall anti-*Mtb* immunity. The exhaustion of CD8^+^T cells diminish their ability to control *Mtb* infection and impede complete clearance of *Mtb*. However, it may also play a critical role in preventing the progression of chronic *Mtb* infection, and anti-PD-1-based therapy needs to be used cautiously in patients with cancer with a history of *Mtb* exposure. Additionally, further research is needed to explore the therapeutic effects of PD-L1 in the treatment of TB. The impact of CD8^+^T cell exhaustion in the *Mtb* infection is multifaceted, not simply beneficial or detrimental. Though the exhausted phenotype of *Mtb*-specific CD8^+^T cells can be restored by certain drugs *in vitro* experiments, it is crucial to observe their impact on disease progression under the complex microenvironment *in vivo* experiments.

In single-cell transcriptomic analysis, *Mtb*-specific T_SCM_ cells possess unique phenotypic and functional profiles that share more similarities with bulk T_CM_ and effector T cells (T_EFF_ cells) than bulk T_SCM_ cells. This suggest that T_SCM_ are exposed to chronic antigen stimulation in *Mtb* infection (Mpande et al., 2018). A functionally impaired and exhausted state of T_SCM_ cells may manifest in *Mtb* infection, similar to what is observed in HIV infection. Recently, a study indicated that HLA-E-restricted *Mtb*-specific T_SCM_ cells are lost during *Mtb* infection and do not fully recover following anti-TB treatment, likely due to infection-induced cellular exhaustion (Azgomi et al., 2022). Studies have shown that there is a parallel differentiation program for human CD8^+^T_SCM_ cells. Stem-like T cells (T_STEM_) and progenitor exhausted-like T cells (T_PEX_) were two clonally, epigenetically and transcriptionally distinct subsets of T_SCM_ and committed to parallel differentiation programs. Acute viral infections would preferentially generate antigen-specific T_STEM_ cells, whereas chronic viral infections would preferentially generate antigen-specific T_PEX_ cells. These subsets were defined by core transcriptional signatures that could be distilled phenotypically into simple profiles, namely CCR7^+^PD-1^-^TIGIT^-^ (T_STEM_) and CCR7^+^PD-1^+^TIGIT^+^ (T_PEX_). T_PEX_ cells are functionally inferior to T_STEM_ and committed to a terminally dysfunctional state but expressed memory-like features (Galletti et al., 2020). In *Mtb* infection, T_SCM_ cells may primarily exist in the form of T_PEX_ cells. Findings from animal models support this hypothesis, showing increased expression of GZMK on peripheral stem cell-like T cells in rhesus macaques infected with *Mtb* (Foreman et al., 2023). According to recent insights into T_SCM_ cells differentiation programs, GZMK expression is a key feature of T_SCM_ cells differentiation towards a functionally exhausted lineage (Galletti et al., 2020), suggesting that T_SCM_ cells may predominantly exist in an exhausted state during *Mtb* infection.

**Figure 2:**
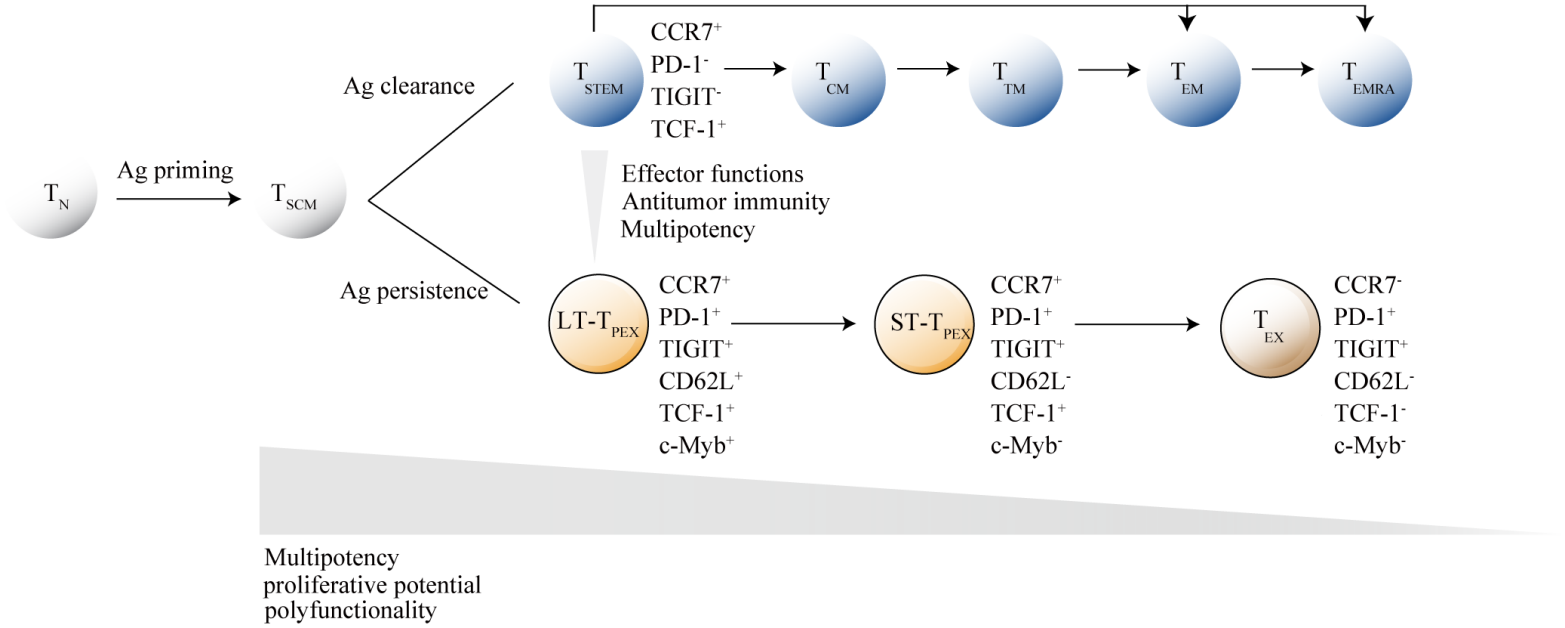
Hierarchical model of memory stem T cells differentiation. After antigen stimulation, naive T cells (T_N_ cells) gradually differentiate into memory T cell subsets, with memory stem T cells (T_SCM_ cells) at the apex of the memory T lymphocyte system. Under different antigen stimulation conditions, T_SCM_ cells develop into either functional T cells or exhausted T cells. When the antigen is cleared, activated T cells differentiate into central memory T cells (T_CM_ cells), transitional memory T-cells (T_TM_ cells) or effector memory T cells (T_EM_ cells), and ultimately into terminally differentiated effector T cells (T_EMRA_). When the antigen persists, T_SCM_ cells differentiate into terminally exhausted T cells (T_EX_) through CD62L^+^ long-term precursor exhausted T cells (LT-T_PEX_) and CD62L^-^ long-term precursor exhausted T cells (ST-T_PEX_). T cell subsets are distinguished by the combinatorial expression of key surface markers. The inhibitory receptor markers TIGIT and PD-1 are crucial for distinguishing between T_SCM_ cells and T_PEX_ cells, while CD62L^+^ and transcription factor c-Myb are the primary markers for identifying different levels in the exhaustion developmental branch. As T_N_ cells gradually differentiate into their terminal states, they lose specific functions.

Akin to adult stem cells, precursor exhausted T cells are hierarchically organized. Developmental trajectory for T_PEX_ cell originates from long-lived CD62L^+^CD8^+^stem-like T cells, which are at a hierarchically superior level compared with their CD62L^-^ counterparts. From CD62L^+^T_PEX_ cells to CD62L^-^T_PEX_ cells to terminally exhausted T cells (T_EX_ cells), a progressive loss of multipotency and repopulation capacity were observed (Tsui et al., 2022) (**Figure 2**). Existence of T_STEM_ and T_PEX_ have been proved in the human CD8^+^ memory T cell pool, and further research is needed to deeply understand of CD62L^+^ stem-like T cells biology and identification of their human counterpart.

## 5 Two-punch attack on CD8^+^T_SCM_ cells in HIV/*Mtb* co-infection

The percentage of *Mtb*-specific CD8^+^T cells identified by tetramers was significantly higher in the circulation of patients with HIV/*Mtb* co-infection compared to those with *Mtb* mono-infection (Manna et al., 2020). What’s more, *Mtb*-specific CD8^+^T cells exhibit further impairment of proliferative capability in co-infection (Manna et al., 2020; Kalokhe et al., 2015). It is possible that HIV- and *Mtb*-driven antigenic stimulation jointly determines the acquisition and maintenance of dysfunctional, exhausted-like traits in *Mtb*-specific CD8^+^T cells. Indeed, previous studies reported that PD-1 was significantly increased on *Mtb*-specific CD8^+^T cells in HIV/*Mtb* co-infection compared to *Mtb* mono-infection, with decreased expression of CD107a, IFN-γ and perforin, furthermore, level of PD-1 expression was associated with reduced IL-2 production capacity (Amelio et al., 2019; Kalokhe et al., 2015; Tan et al., 2023). Differences exist not only between patients with HIV/TB and TB, but also between those with HIV/LTBI and LTBI. However, the relevant studies are mainly conducted in ART-naïve individuals, they cannot explain why PLWH with sustained viral suppression still have a higher risk of *Mtb* infection. To prove whether dysregulation of *Mtb*-specific T cell functional homeostasis induced by HIV infection can potentially enhance the onset of TB in LTBI subjects, it is imperative to investigate that in long-term ART-treated aviremic HIV-infected patients.

*Mtb*-specific CD8^+^T cells identified by with ESAT-6 and/or CFP-10 peptide pools stimulation assays were mostly represented by T_EM_ cells in TB patients (Rozot et al., 2013). Another study showed that mean 45% of *Mtb*-specific CD8^+^T cells restricted by HLA-E were composed of T_EMRA_ cells in patients with active TB disease, and 70% of *Mtb*-specific CD8^+^T cells restricted by HLA-E in HIV/*Mtb* co-infected patients were composed of T_EMRA_ cells. Thus, *Mtb*-specific CD8^+^T cells response in HIV/*Mtb* co-infection appears to be largely dominated by a differentiated effector-memory profile (Manna et al., 2020). This indicates that the persistent stimulation by HIV and *Mtb* antigens enhances the terminal differentiation of CD8^+^T cells, leading to a further decrease in the proportion of T_SCM_ cells.

**Figure 3:**
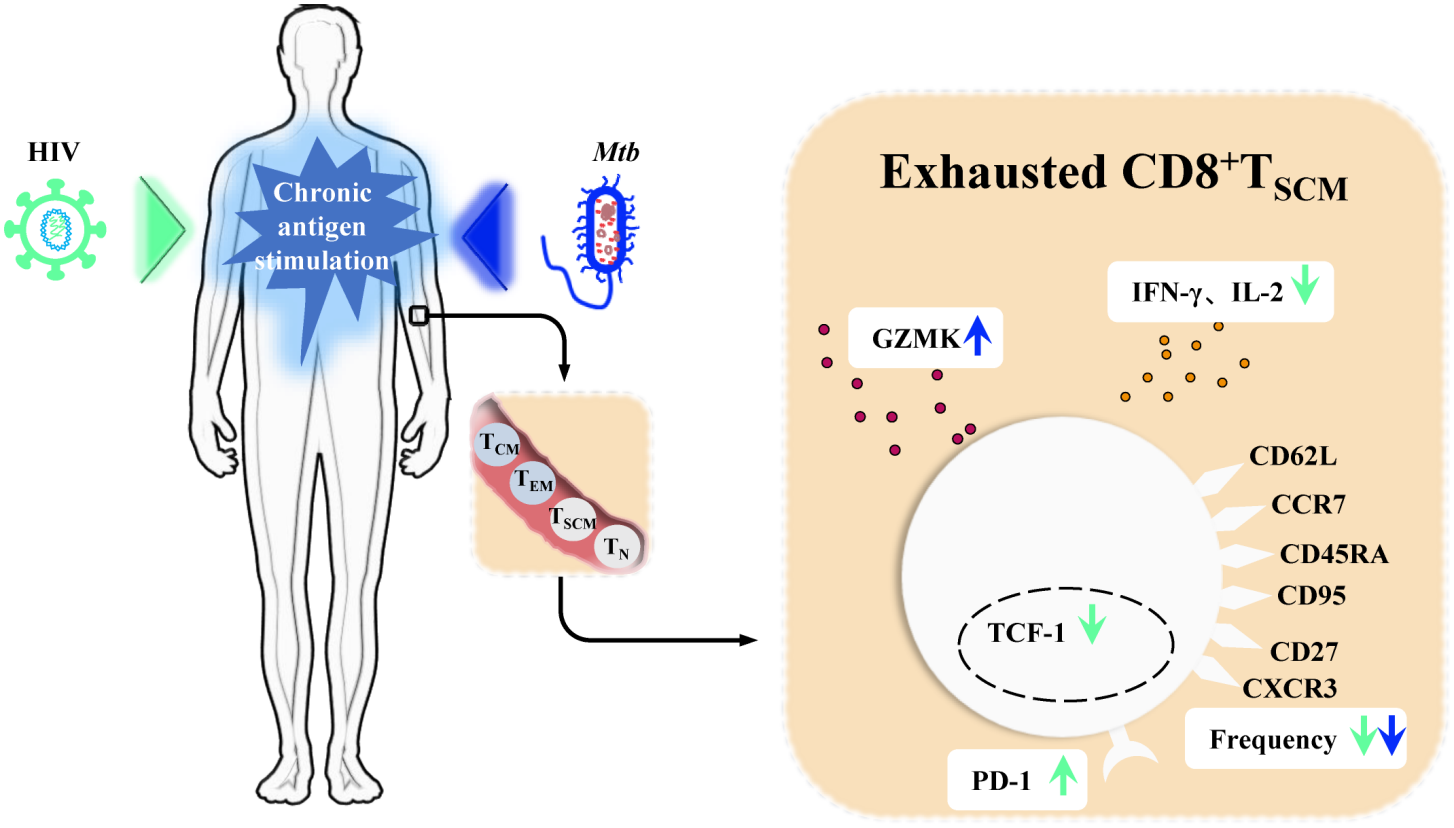
CD8^+^T_SCM_ cells in chronic Human immunodeficiency Virus (HIV) infection and *Mycobacterium tuberculosis* (*Mtb*) infection. In chronic Human immunodeficiency Virus (HIV) infection and *Mycobacterium tuberculosis* (*Mtb*) infection, persistent antigenic stimulation drives memory stem T cells (T_SCM_ cells) toward terminal exhaustion. In chronic HIV infection, CD8^+^T_SCM_ cells exhibit increased expression of the inhibitory receptor PD-1, reduced levels of IFN-γ and IL-2, and decreased expression of the self-renewal marker TCF-1. Staining and quantification of their characteristic surface markers further reveal a decline in their frequency. During *Mtb* infection, CD8^+^T_SCM_ cells show a decreased frequency, as seen in HIV infection, but exhibit elevated expression of GZMK.

During *Mtb* infection, T cell metabolism and function deteriorate over time. This is manifested by bioenergetic insufficiency in *Mtb*-specific T cell populations, mitochondrial dysfunction, and restricted cytokine production, all early signs of T cell exhaustion (Russell et al., 2019). In HIV/*Mtb* co-infected individuals, this deterioration is exacerbated. Compared to patients with TB alone, markers of T cell exhaustion, such as PD-1 expression, are further elevated in *Mtb*-specific T cells of HIV/*Mtb* co-infected individuals. This is accompanied by declines in cytotoxicity and proliferation functions (Manna et al., 2020; Amelio et al., 2019). Similar differences are observed between LTBI with and without HIV infection (Manna et al., 2020; Amelio et al., 2019). In both HIV and Mtb mono-infections, CD8^+^T_SCM_ cells undergo chronic stimulation, leading to exhaustion. This is characterized by increased expression of co-inhibitory molecules (PD-1) and exhaustion markers specific to T_SCM_ cells (GZMK), diminished cytokine secretion capacity (IFN-γ, IL-2) and self-renewal marker TCF-1, and a reduced proportion of these cells (Ribeiro et al., 2014; Vieira et al., 2023; Foreman et al., 2023; Tuluc et al., 2017; Takata et al., 2022; Vigano et al., 2015) (**Figure 3**). In the context of HIV/*Mtb* co-infection, the chronic stimulation from dual pathogens is likely to further aggravate these effects. However, current analyses of T cells in human *Mtb* infection and HIV/*Mtb* co-infection have primarily relied on traditional markers such as CD45RA and CD62L or CCR7. These markers do not effectively distinguish T_SCM_ cells, leaving the phenotype, functional differences, and mechanisms involved in T_SCM_ cells during HIV/*Mtb* co-infection remain unresolved questions, requiring further research for exploration.

## 6 Potential mechanisms of interaction between HIV/*Mtb* co-infection and CD8^+^T_SCM_ cells

CD8^+^T cell-intrinsic IL-27 signaling safeguards the ability of TCF1^hi^ cells to maintain proliferation and avoid terminal differentiation or programmed cell death. Mechanistically, IL-27 endowed rapidly dividing cells with IRF1, a transcription factor that was required for sustained division in a cell-intrinsic manner (Huang et al., 2019). Single-cell transcriptomics and epigenomics approaches revealed that BACH2 establishes the transcriptional and epigenetic programs of stem-like CD8^+^T cells. BACH2 overexpression enforced stem-like cell fate, whereas BACH2 deficiency impaired stem-like CD8^+^T cell differentiation. BACH2 suppressed the molecular program driving terminal exhaustion through transcriptional repression and epigenetic silencing (Yao et al., 2021). NR4A1 was previously found to be important for T cell dysfunction. Hao et al. further demonstrate that NR4A1 regulates T_PEX_ cells development and maintenance in the tumor microenvironment. NR4A1 inhibits effector cytokine production and fosters accumulation of T_PEX_ cells by directly stimulating T_PEX_-related genes while repressing genes associated with terminal exhaustion (Tsui et al., 2022). FOXP1, a hub in the stem-like network, promoted expansion and stemness of chimeric antigen receptor (CAR)-T cells and limited excessive effector differentiation. In the effector network, KLF2 enhanced effector CD8^+^T cell differentiation and prevented terminal exhaustion (Zhu et al., 2024). In the hierarchical fashion of precursor exhausted T cells. c-Myb has a critical role in restraining exhausted T cell differentiation. The transcription factor MYB is not only essential for the development of CD62L^+^T_PEX_ cells and maintenance of the antiviral CD8^+^T cell response, but also induces functional exhaustion and thereby prevents lethal immunopathology (Tsui et al., 2022). Although many studies have focused on elucidating the mechanism of CD8^+^T_SCM_ cell differentiation since the discovery of parallel differentiation programs, the majority of these studies have been conducted in animal models. Due to significant physiological and immunological differences between animals and humans, the findings may not be directly applicable to human biology. Currently, a comprehensive understanding remains elusive.

**Figure 4:**
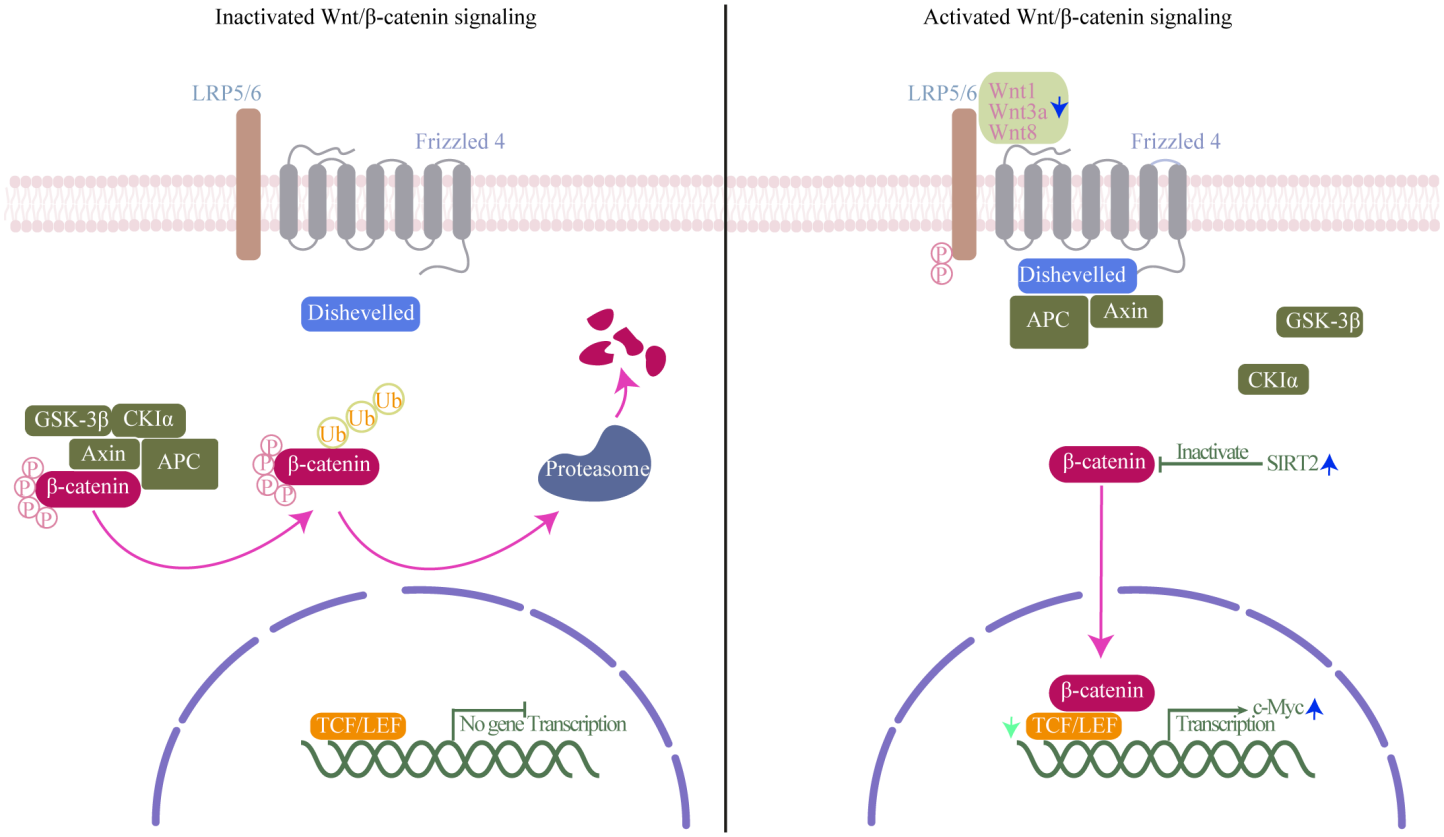
Wnt/β-catenin signaling mechanism. Left (inactivated Wnt/β-catenin signaling): In the absence of Wnt ligands, β-catenin interacts with a degradation complex composed of axis inhibition protein (axin), adenomatous polyposis coli (APC), glycogen synthase kinase-3β (GSK-3β), and casein kinase 1α (CK1α). Within this complex, β-catenin undergoes phosphorylation by GSK-3β and CK1α, followed by ubiquitination and degradation in the proteasome. Right (activated of Wnt/β-catenin signaling): Wnt ligands bind to the frizzled receptor and the co-receptor low-density lipoprotein receptor-related protein (LRP). This interaction induces the phosphorylation of LRP by GSK-3β and CK1α, leading to the recruitment of axin and disheveled to the LRP/Frizzled receptor complex, thereby releasing β-catenin. Subsequently, β-catenin accumulates in the nucleus, where it binds to lymphocyte enhancer factor-1 (LEF1) and T cell factor (TCF) to initiate the transcription of specific genes. Green arrow, TCF expression decreases in Human immunodeficiency Virus (HIV) infection; blue arrow, Wnt1, Wnt3a, and Wnt8 are downregulated, and class III HDAC SIRT2 and transcription factor c-Myc expression increases in *Mycobacterium tuberculosis* (*Mtb)* infection.

Wnt/β-catenin signaling pathway, classically considered necessary for cell differentiation, effector functions and migration, is the canonical Wnt signaling pathway and the best understood and characterized pathway of Wnt signaling (Gattinoni et al., 2010). Activation of Wnt/β-catenin signaling pathway results in β-catenin accumulation and translocation to the nucleus where it drives the expression of T-cell factor/lymphoid enhancer-binding factor (TCF/LEF)-dependent genes, which are important for self-renewal capacity of CD8^+^T_SCM_ cells (Lin et al., 2016) (**Figure 4**). CD8^+^T cells have both a cytolytic effect on infected cells before SIV integration, and a direct, non-cytolytic effect by suppressing viral production (Policicchio et al., 2023). Wnts expressed by CD8^+^T cells can mediate CD8^+^T cell noncytolytic anti-HIV-1 activity by canonical Wnt signaling in HIV-infected recipient cells (Wallace et al., 2020). Influenced by HIV, concomitant loss of active Wnt/β-catenin genetic signature at the single-cell level was observed during HIV infection (Kared et al., 2020). Indeed, decreased expression levels of TCF-1 and loss of CD8^+^T_SCM_ cells have been proved in HIV infection (Takata et al., 2022). Furthermore, Similarly, in *Mtb* infection, key genes of Wnt/β-catenin signaling were impaired in blood cells of patients with severe pulmonary TB, furthermore, β-catenin expressions in CD8^+^T cells were significantly decreased in patients with severe pulmonary TB compared with those in mild diseases (Fan et al., 2017; Xiong et al., 2021). SIRT2, a class III HDAC, is overexpressed in *Mtb*-specific CD4^+^T cells. Inhibition of SIRT2 enhances could enhances CD4^+^T_SCM_ cells response by activating b-catenin, and finally enhances the BCG vaccine efficacy during primary infection and TB recurrence (Bhaskar et al., 2023). As counterparts to CD4^+^T cells, SIRT2 regulation has also been proven in CD8^+^T cells (Jiang et al., 2020). Similar effects may occur in CD8^+^T_SCM_ cells under SIRT2 inhibition. same effect may appear in CD8^+^T_SCM_ under inhibition of SIRT2. Using single cells RNA sequencing and high-dimensional flow cytometry, Kared et al. demonstrate that T_SCM_ heterogeneity results from differential engagement of Wnt signaling. In humans, aging is associated with the coupled loss of Wnt/β-catenin signature in T_SCM_ cells (Kared et al., 2020). It hints that HIV and *Mtb* infection may cause a certain of caused a certain degree of immunosenescence, leading to disruptions in Wnt/β-catenin signaling, which in turn causes dysfunction in the immune function of CD8^+^T_SCM_ cells. And it is reasonable to assume that Wnt/β-catenin signaling pathway deteriorates in HIV/*Mtb* co-infection than in mono-infection. In recent years, with the emergence of immunotherapy, the indispensable role of Wnt in regulating T cell development and differentiation has been recognized (Pai et al., 2017). Modifying the activity of Wnt/β-catenin signaling is an attractive therapeutic approach for infectious diseases. However, given the limited number of relevant studies, the regulation mechanism and alteration require further investigation and validation through more comprehensive omics studies.

## 7 Clinical applications targeting CD8^+^T_SCM_ cells

### 7.1 Vaccine

Many T cell subtypes have been shown to be key responders to various pathogen infections and are utilized to predict vaccine effectiveness. For instance, tissue-resident memory T cells (T_RM_) in the respiratory tract play a crucial role in limiting the severity of SARS coronavirus infections (Zheng and Wakim, 2022; Buggert et al., 2023b). Consequently, the induction of cytokine-secreting T_RM_ cells has been widely employed to forecast improved clinical outcomes for patients and enhanced protective efficacy for vaccine recipients (Buggert et al., 2023a; Zheng and Wakim, 2022). While the primary assurance of infection prevention lies in the induction of neutralizing antibodies, the cytotoxic CD8^+^T cell responses are of particular importance in the elimination of pathogens (Plotkin, 2008). Report has shown the persistence of yellow fever specific CD8^+^T_SCM_ cells for 25 years post vaccination (Marraco et al., 2015). Among HPV-specific CD8^+^T cells induced by vaccine, CD8^+^T_SCM_ cells were found to be stronger and long-term anti-tumor function, highlighting its crucial role in the process of vaccine efficacy (Zhang et al., 2020). However, the efficacy of the vaccine in HIV-infected patients may be compromised. Impaired primary responses of CD8^+^T cells to vaccination exist in older individuals, and many of the immune alterations in HIV-infected individuals resemble the process of immune aging, which is characteristic of old age (Schulz et al., 2015; Chauvin and Sauce, 2022). In elderly individuals, BCG vaccination induced diminished frequencies of CD8^+^T_N_ and T_SCM_ cells (Kumar et al., 2021). The loss of CD8^+^T_SCM_ cells may also occur in HIV patients given BCG vaccination. Of note, referring to the impact of aging on CD8^+^T_N_ cells (Gustafson et al., 2019), CD8^+^T_SCM_ cells may also undergo phenotypic, functional, transcriptional, and epigenetic deterioration in HIV infection. Impairment of CD8^+^T_SCM_ cells immune function potentially account for a reduction in vaccine effectiveness in HIV-infected patients given *Mtb* vaccine. Fortunately, vaccines combined with adjuvant formulations that stimulate the generation of CD8^+^T_SCM_ cells are promising to enhance the effectiveness of the vaccine. Generation of CD8^+^T cells response is regulated by T cell receptor (TCR) signaling, and investigation of TCR downregulation and manipulation of TCR signaling strength may help design vaccines to elicit CD8^+^T_SCM_ cells, capable of surviving antigen restimulation to generate antiviral effects (Wu et al., 2017). Moreover, in the settings of circulating and evolving viruses, CD8^+^T_SCM_ cells is a remarkably stable marker of long-term protection against evolving pathogen, thus, measuring vaccine-induced T_SCM_ cells may be more accurate to predict the effectiveness of vaccines (Aleksova et al., 2023).

### 7.2 CAR-T

Cumulating evidence in mice indicates that the infusion of less-differentiated T cells results in greater cell expansion, persistence in adoptive immunotherapy (Hinrichs et al., 2009; Sommermeyer et al., 2016; Klebanoff et al., 2016). Quiescent memory T cells seem to be more susceptible to lentiviral transduction than their naive counterparts (Ghassemi et al., 2022). Thus, compared with T_N_ and other memory subsets of T cells, T_SCM_ cells type is an ideal cell population to improve CAR-T cell therapy’s time-dependent efficacy and stability for its extreme longevity, the robust proliferative potential and the capacity to reconstitute a wide-ranging diversity of the T cell compartment (Ahmed et al., 2016). CAR-modified CD8^+^T_SCM_ cells mediated superior and durable responses in anti-tumor roles, CD8^+^T_SCM_ cells might also provide an attractive approach for immunotherapy in the setting of chronic infection. However, T cell immunotherapy targeting T_SCM_ cells is limited by the relatively small proportion of these cells. In peripheral blood, T_SCM_ cells account for 2%∼4% of CD8^+^T cells (Lu et al., 2016). Many new regulators of CD8^+^T_SCM_ cells have been found, such as gene encoding transcriptional repressor BACH2 (Yao et al., 2021), IL-33 (Marx et al., 2023), TGF-β (Hu et al., 2022), CXCR3 (Bangs et al., 2022), and HMGB2 (Neubert et al., 2023), which sheds light on future interventions that harness the differentiation of therapeutic T cells to treat chronic infection. IL-7 and IL-15 have been implicated in the generation and maintenance of T_SCM_ cells (Cieri et al., 2013). Recently, a simplified protocol enabling efficient derivation of gene-modified CD8^+^T_SCM_ cells from CD8^+^T_N_ cells by culturing with IL-7 and IL-15 was presented which may facilitate improved adoptive immunotherapy (Kranz et al., 2022). A mechanistically novel peptide agonist of the IL-7 receptor, MDK-703, could induce pronounced expansion of memory T-cells, particularly the population of T_SCM_ cells (Dower et al., 2023). The Wnt/β-catenin signaling pathway is one pathway which is likely to be involved in influencing whether T_SCM_ cells undergoes self-renewal or differentiation (Gattinoni et al., 2009). Treatments such as β-catenin inhibitors would be useful for assisting in the treatment of HIV-1, acting as a prompt for the formation of CD8^+^T_SCM_ cells (Denk et al., 2022).

## 8 Conclusion

Accumulating evidence has illuminated the significant role of CD8^+^T cells in both HIV and *Mtb* infections. Moreover, more pronounced alterations in CD8^+^T cells during co-infection have been observed, highlighting close associations with disease progression. Delving into the evolutionary characteristics, mechanisms, and functions of CD8^+^T_SCM_ cells in co-infection contributes to a deeper understanding of immunological mechanisms. In the differentiation process of CD8^+^T cells, CD8^+^T_SCM_ cells are at the apex in the hierarchical system of memory CD8^+^T lymphocytes, holding potential implications for the development of immunotherapies and vaccines. While research about CD8^+^T_SCM_ cells in HIV/*Mtb* co-infection is currently limited, noteworthy changes identified in existing articles underscore the need for further studies to elucidate these mechanisms.
